# Supplementary material for: Are carbon emissions trading and green financial instruments synergistic? -Comprehensive quantitative research based on content analysis
Source: PLoS One. 2024 Mar 7;19(3):e0298601. doi: 10.1371/journal.pone.0298601 (PMC10919601; doi:10.1371/journal.pone.0298601)
Supplement: S1 Data — (ZIP) [file pone.0298601.s001.zip › Data/地级市Z_F工作报告.zip!/╣π╢1⁄2/╘╞╕í╩╨/└┤╫╘╣1⁄2╓┌║┼í╛┬φ┐╦╩2╛▌═°í┐_2007.docx]

各位代表：

我代表市人民政府向大会作政府工作报告，请予审议，并请政协委员和其他列席人员提出意见。

过去三年工作的回顾

2004年换届以来,市政府在省委、省政府和市委的正确领导下，在市人大及其常委会和市政协的监督、支持下，高举邓小平理论和“三个代表”重要思想伟大旗帜，认真落实科学发展观，围绕市第三次党代会制定的“东渐西联、优内拓外、错位竞争、统筹并进”发展战略，以加快经济发展为中心，坚定不移走工业化、城镇化和农业产业化发展路子，加快工业发展步伐，加大招商引资和项目建设力度，全市经济实现了加快发展；大力推进科技、教育、文化、卫生、体育等各项社会事业发展；扎实推进民心工程建设，着力解决民生问题，努力构建和谐云浮。经过全市人民的共同努力，全市经济社会实现平稳较快发展，取得显著成绩，圆满完成了市三届人大一次会议提出的（本届政府前三年）各项目标任务。

——紧紧抓住经济发展这个中心，总体经济实力明显增强。2006年，预计全市实现生产总值245.4亿元，比上年(下同)增长13.8%，比2003年增长41.6%；万元生产总值能耗1.37吨标准煤，降低3.5%，比2003年降低5.7%；工业总产值286.2亿元，增长23.2%，比2003年增长78.1%；农业总产值127.4亿元，增长6%，比2003年增长19.3%；社会消费品零售总额70.1亿元，增长17.6%，比2003年增长64.9%；全社会固定资产投资总额121.4亿元，增长13.1%，比2003年增长104.5%；外贸出口总额5.6亿美元，增长30.7%，比2003年增长98.2%；实际利用外资0.58亿美元，增长52.6%，比2003年增长815%；地方财政一般预算收入11.39亿元，增长22.06%，比2003年增长93.2%；城镇居民人均可支配收入10317元，增长10.4%，比2003年增长34.4%；农村居民人均年纯收入4493元,增长6.3%，比2003年增长18.3%。

——紧紧抓住发展工业这个重点，工业发展明显加快。抓住国内外产业转移机遇，发挥云浮资源优势，把加快工业发展作为经济工作的重中之重，重点抓现有几大工业行业做大做强、发展产业集群、承接产业转移、招商引资、工业园区和项目建设，以及出口带动等，有力地促进了全市工业加快发展，全市工业对经济增长的拉动力明显增强。2006年，全市规模以上工业总产值197.3亿元，比2003年增长88.2%；重工业产值159.8亿元，增长100%；轻工业产值126.4亿元，增长56.4%；工业增加值占生产总值比重提高7.7个百分点；工业经济效益提高7.6个百分点。几大工业行业中，机械电子年均增长44.5%、硫化工年均增长32.5%、不锈钢制品年均增长30.4%、纺织服装年均增长22.9%、石材年均增长22.7%、水泥年均增长22.2%、电力年均增长20.6%。三年新增国家免检产品2个、广东省名牌产品6个、广东省著名商标12个；新增投产500万元以上生产性项目320个，实际投资总额105亿元。云硫集团年产40万吨硫酸扩建项目可行性研究已获省审批，云浮电厂三期工程已获国家发改委批复同意开展前期工作；华润西江发电厂已列入省“十一五”规划，粤泷电厂二期正在争取省支持；西江内河核电项目郁南罗旁厂址初可已经国家电力规划设计院评审通过，评为西江内河核电优选厂址，即将进入可行性研究。

——紧紧抓住农业和农村经济这个基础，农村经济水平明显提高。努力改善农村生产生活条件，大力推进农业产业化经营，加快发展特色农业和劳务经济，战胜了西江超百年一遇洪水、台风“派比安”等自然灾害的袭击，成功防控了禽流感疫情，较好地实现了农业增产、农民增收和农村稳定。2006年，全市粮食播种面积238万亩，总产量96.9万吨，连续三年超额完成省下达的任务；2004年、2005年粮食工作考评均获省奖励，罗定市被评为2005年度全国粮食生产先进县。全市农业产业化经营组织发展到146个，比2003年增加51个；年销售收入120亿元，增长63.3%；带动农户23.8万户，增长30%；户均年增收4700元，增长18.7%。11个苦旱镇农业龙头企业发展到19家，带动农户3.4万户。特色农业生产基地650万亩，比2003年增长10%；水果产值增长62.1%；畜牧业产值增长38.5%、渔业产值增长18.2%。肉桂、松脂、竹木等林产品深加工取得新发展。三年累计造林68.6万亩，郁南县被评为全国绿化模范县，与罗定市被评为省级林业生态县（市）。2006年，全市农村固定资产投资总额50.4亿元，比2003年增长72.5%；农村消费品零售总额30.7亿元，增长64.4%；外出农民工46.6万人，增长11%；劳务总收入增长29.4%。

——紧紧抓住发展县域、镇域经济这个关键，县域、镇域经济不断壮大。坚持“强市之基在于强县，强县之基在于强镇”的发展理念，加快工业化、城镇化和农业产业化步伐，利用资源优势，发展优势产业、优势企业和优势产品；组织县、镇领导干部到发达地区参观学习和承接产业转移，实施经济责任考核和行政激励措施，每年召开发展镇域经济现场会，调动各级领导干部抓发展的积极性，推动了县域、镇域经济加快发展。城区工业和第三产业蓬勃发展。2006年，城区实现工业总产值比2003年增长124.2%、服务业增长61.1%、地方财政一般预算收入增长87.5%。县域工业发展明显加快，特色产业规模继续扩大，石材、不锈钢制品产业被确认为省级产业集群；农业产业化经营水平、经济效益、社会效益不断提高，部分农业龙头企业加快融入农产品深加工等二、三产业，成为推动县域、镇域经济发展的重要力量；城镇化加快推进，城镇建设步伐加快，面貌明显改善。全市县域生产总值比2003年增长50.9%，工业总产值增长95.2%，税收收入增长94.5%；县（市、区）地方财政一般预算收入全部实现超亿元，其中云安、郁南、新兴和罗定2005年度财政综合增长率分别排在全省67个县（市）第4位、第7位、第 9 位和第36位，均获得省财政转移支付激励奖。全市镇（街）生产总值比2003年增长52.8%，工业总产值增长102.9%，税收收入增长102.3%。民营经济成为县域、镇域经济发展的主体。全市个体私营企业发展到4.9万户, 比2003年增长28.9%，其中私营企业1819户，增长84.3%。民营工业增加值60.1亿元，增长103.1%。

——切实加大服务业发展力度，服务业呈现快速发展态势。大力推进商贸、旅游、餐饮、房地产、运输、信息、物流、中介、金融、保险等行业发展，进一步优化经济结构、促进就业、繁荣市场，拉动经济快速增长。启动了“创建中国优秀旅游城市”工作，开发了罗定龙湾生态瀑布、郁南大湾古民居、新兴东成和水台温泉等旅游项目，完善了蟠龙洞、龙山等风景区建设。2006年，接待旅客人数307.7万人次，比2003年增长96%；旅游收入24.5亿元，增长101%；货物周转量19.9亿吨公里，增长25.8%；港口货物吞吐量609万吨，增长23.8%；邮电通信业务总量18.3亿元，增长92.6%；金融机构本外币各项存款余额251.9亿元，增长47.1%；保险机构保费收入3.5亿元，增长59%。三年共投入12.7亿元，建成住宅小区23个、建筑面积 148.2万平方米；投资15亿元，新增商贸服务业项目55个；建设和改造省级以上“万村千乡市场工程”农家店254家。

——切实加大外向拉动力度，利用外资和进出口取得优异成绩。各级政府主动搭台，积极组织企业参加各种经贸活动，充分发挥驻珠三角招商联络处等招商网络的作用和我市的产业、资源优势，吸引产业转移项目和外资到我市落户；积极改善出口通关和物流运输条件，推进保税仓建设，组织企业“走出去”，扩大对外经贸。三年共签订投资500万元以上生产性项目448个，金额246亿元，其中已落实401个，投资总额177亿元。2005年实际利用外资增速排全省地级以上市第2位，2006年排第3位。2006年，一般贸易出口1.2亿美元，比2003年增长143.3%；加工贸易出口3.9亿美元，增长71.8%。其中机械电子出口增长227.5%、石材出口增长140.2%。与我市有贸易往来的国家和地区发展到108个，比2003年增加20个。我市与保加利亚韦林格勒市初步确定了友好城市关系，与挪威王国拉尔维克市、斯洛伐克特伦钦地区建立了合作交流关系；与贵州省黔西南州结为友好市（州）。中材、华润、亚铝等知名企业陆续进入我市投资发展。2005年，我市首次获得省实际利用外资完成进度贡献奖、新增世界500强跨国公司投资奖和口岸大通关建设奖。

——切实加大承接产业转移力度，促进工业发展步伐加快。认真落实省委、省政府关于做好承接产业转移的重要决策，将承接产业转移工作作为招商引资工作的重中之重，加大力度，努力营造优良的投资环境，积极做好各项服务工作，主动承接产业转移。2005年以来，我市与佛山市建立了共建产业转移园区工作联席会议制度，先后6次赴佛山市专门研究有关产业转移事项，佛山市政府也组织企业家来我市考察投资环境。我市各级主要领导亲自带队，先后27次组团到佛山、中山、东莞等地开展产业转移承接活动。至2006年底，全市共引进来自珠三角地区的产业转移项目314个，计划投资总额196亿元。其中与佛山市签约项目79个，投资额60亿元。我市与佛山市共建的都杨和新成工业园区获省批准成为产业转移工业园区，两个园区已投入建设资金1.73亿元，已落实7个项目签约入园区发展。郁南县与中山市签订了共建产业转移园区协议，罗定市与东莞市电子行业协会签订了合作建设双东环保工业园协议。云安六都工业园以循环经济特色吸引了一批产业转移项目。

——切实加大基础设施建设力度，城建、交通、水利、环保建设成绩显著。市区三年共投入9.2亿元，竣工工程74项，建成新世纪大道、金山大道等基础设施；建成电信大楼、国税大楼、档案馆、群众艺术馆、市妇女儿童活动中心等办公文化场所；建成凯旋大酒店、风情步行街等一批服务配套设施；市委党校新校区、市中级法院审判大楼、市图书馆、博物馆等工程即将竣工，使市区市政设施进一步完善，城市品位、档次进一步提高。各县（市）累计共投入16.9亿元，建设村镇基础设施项目5451个，其中投入中心镇建设9.2亿元。城镇化水平达43%，比2003年提高2个百分点。三年共投入交通基础设施建设34.8亿元，完成公路建设改造2082公里，全市通车公路总里程6805公里（含村道），实现了县到镇通三级以上公路，镇到行政村基本通硬底化公路。广梧高速公路一期马安至河口段和省道280线、369线建成通车，广梧高速公路二期河口至平台段和罗定至岑溪铁路全面开工建设，大环市路云安至高要大湾段和云城至高村段也即将全面开工建设，云浮至广西岑溪高速公路（粤境段）、云浮港六都四围塘码头和都骑通用码头前期立项工作顺利推进。供电、环保、水利等设施建设加快。三年共投入7.6亿元，新建(扩建)35千伏以上变电站13座，保障了城乡居民和企业用电；投入9.7亿元，建成市城区一期和罗定市一、二期生活污水处理厂以及城区生活垃圾卫生填埋场，对水泥、石材等行业和西江等水域进行环境综合整治，市区每月每平方公里的降尘量由2003年的7.8吨降至4.6吨，大气质量达到国家二级标准，饮水水源水质达国家标准，地表水环境质量达到功能要求；投入8.07亿元，动工建设10宗城乡水利防灾减灾工程，完成1054宗其它水利工程项目建设，全面提高了大江大河、中小型水库防灾抗灾能力。

——着力加强财税工作，财税、金融保持健康较快发展。坚持一手抓经济发展，培植税源；一手抓依法治税，强化征管，抓好非税收入和收支两条线管理，积极盘活政府资源，保证了财税收入平稳较快增长。2006年，全市国税收入11.4亿元，比2003年增长74%；地税收入9.6亿元，增长85.1%。初步建立了公共财政框架，保障了工资发放、社会事业发展和机关正常运作，全市财政（含上级补助）对新农村建设、农村“一保五难”和重点保障等的投入三年累计达25亿元。金融服务经济有新发展，新引入国家开发银行等金融机构开展业务。成立中小企业信用贷款担保公司，积极利用资本市场，扩大融资渠道。全市金融机构三年累计发放贷款158.6亿元，其中工商企业短期贷款36.5亿元、农业短期贷款26.6亿元。切实抓好地方金融资产追收工作，三年共收回城信社、农金会资产4174万元。农村信用社呆帐清收和不良贷款压降工作进展顺利。

——着力加强社会建设，各项社会事业和精神文明建设取得新进步。大力推进科技、教育、文化、卫生、体育等各项事业建设，深入开展群众性精神文明创建活动，提高城乡文明水平，促进经济社会协调发展。省级以上科技创新载体发展到63 个，比2003年增加42个；科技进步对经济增长贡献率达48%，提高2个百分点。巩固提高九年义务教育，加快发展普通高中教育和职业教育。完成51所老区山区学校改造，新建扩建普通高中学校6所、中等职业学校2所，扩大了罗定职业技术学院和新兴中药学校办学规模，新组建了市技工学校，动工建设市中等职业技术学校。高考上省专科A线人数比2003年增长34.39%，上线率连年高于全省平均水平。“一机两语”特色教育走在全省山区市前列，2005年全省农村中小学“一机两语”教育现场会在我市召开。大力开展群众文化活动，加强南江文化、禅宗文化、石文化等资源的开发利用，加快群众文化基础设施建设，发展文化产业。《六祖惠能》音乐剧成功首演并取得良好社会效应。全面开展新一轮地方志修编工作。大力开展全民健身运动，参加省第十二届运动会金牌和总分分别列第11名和第12名、省第五届残疾人运动会列团体总分第9名、省第三届少数民族传统体育运动会列团体总分第10名，三年累计获得省级以上冠军44个,其中世界冠军和亚洲冠军各1个。进一步完善公共卫生三大体系建设，新建、改造疾病预防控制中心5个、薄弱卫生院7间，创建省级卫生村87条、卫生先进镇3个。规范药品、医疗器械招标，让利群众6700万元。理顺了红十字会体制。我市被授予全省两个创建农村中医工作先进市示范单位之一。2006年全市人口出生率、自然增长率、计划生育率分别为10.85‰、5.61‰和92.02%，县（市、区）全部进入人口与计划生育一、二类工作基础类型管理范围。

——着力解决民生问题，全市社会和谐稳定。坚持以人为本，积极抓好以“一保五难”为重点的各项“民心工程”建设，努力构建和谐云浮。全市征收社保费4.3亿元，比2003年增长47.5%；城镇登记失业率为2.85%；享受低保救济人数增加2.4万人，实现了应保尽保。由政府出资解决了破产、关闭、转制企业1.7万多名退休人员参加住院保险问题。全面解决了18万农村群众的饮水难问题。新型农村合作医疗覆盖率达91.1%，我市双档型农村合作医疗制度荣获中国十大政府创新典型奖。三年帮助2090户受灾全倒户重建家园；建成单亲特困母亲安居房163户；妥善解决全市171户城镇特困户居住问题；改造农村贫困户危房6634户。完成939个行政村公路硬底化改造，占总任务的97.2%；建成1015条生态文明村；开展劳动技能培训10万人次，选送1690名贫困家庭子女参加学历教育；全面落实农村义务教育阶段学生免收学杂费和困难学生减免书杂费政策，减免金额达1.4亿元；清理废止或调整降低涉农收费项目61项，减轻农民负担2800多万元；办理法律援助案件1524件，援助困难群众2200多人。深化非煤矿山、危险化学品、道路、建筑工地的安全生产整治和监督，全面退出爆竹生产行业，安全事故得到有效控制。实行食品药品市场准入，食品药品安全监督管理收到初步成效。加强社会治安综合治理，严厉打击各种刑事犯罪活动，大力开展禁毒和打击“两抢一盗”等专项行动。建立完善了领导接待群众来访、领导包案、信访工作目标管理考核等制度，及时化解各类矛盾纠纷，全市大局稳定。

——着力加强政府自身建设，政务环境明显改善。坚持依法行政，深入开展法制宣传教育，认真贯彻《行政许可法》、《公务员法》和国务院《全面推进依法行政实施纲要》等法律法规。三年共出台政府规范性文件42件，办理行政复议案件94件。坚持和完善向市人大及其常委会报告工作、向市政协通报情况的制度，自觉接受监督。三年共办理人大议案、建议294 件，政协提案219件。加强与工会、共青团、妇联和工商联等群众团体和党外人士的联系。实行重大事项专家咨询、社会听证、决策评估和市政府常务会议集体决策制度。开展企业、群众民主评议政府部门和政风行风热线活动。从2005年起，市政府实行分解年度工作要点、部门负责抓落实机制，厉行抓落实工作作风，促进了各项工作的落实。市直单位全部实行部门预算管理，并开展国库集中支付、财政支出绩效评价试点工作。经济责任审计工作全面铺开。政府采购进一步规范，政府投资工程建设、经营性土地使用权和采矿权有偿出让全部实行招投标管理，国有资产转让全部进场公开交易。清理取消和调整行政审批事项169项，审批事项基本进入政府行政服务中心办理，建成我市电子政务基础平台和行政电子监察系统，对行政审批事项实行实时监控。深化政务、厂务、村务公开工作，积极开展“活力民主，阳光村务”工程试点工作和“十百千万”干部下基层、争当排头兵等活动。

此外，人防工程规划建设、耕地和矿产资源保护、发展规划、物价、粮食、统计、人事人才、编制、知识产权、经济发展研究、民族宗教、档案、民政、双拥、武装、国防动员、民兵预备役、外事侨务、台务、消防、保密、新闻出版、贸促会、信息产业、气象、侨联、工商联、文联、社科联、残联、妇女儿童、慈善会以及驻市外机构等工作也取得了新的成绩。

各位代表, 第三届政府三年来的各项工作目标胜利完成，经济社会发展取得显著成绩。这是在省委、省政府和市委的正确领导下，在社会各界的大力支持下，全市人民奋发努力，积极进取的结果。在此，我代表市人民政府，向全市人民、驻云浮部队官兵表示崇高的敬意！向关心支持云浮建设和发展的香港、澳门特别行政区同胞、台湾同胞和海外侨胞及国际友人表示衷心的感谢！

同时，我们也清醒看到，当前我市经济社会发展仍存在不少制约因素和薄弱环节，主要是:经济总量较小，政府财力薄弱，各项社会事业发展受到制约；工业化程度较低，经济结构不合理；外贸出口和利用外资的规模较小，对经济发展拉动力不足; 交通等基础设施建设滞后，经济社会发展硬环境未得到根本改善；自主创新能力不强，经济增长方式粗放，资源、环境保护的压力较大；农民收入增长缓慢，持续增收难度较大；安全生产、社会治安、食品、药品安全等领域存在一些隐患；政府职能和机关作风仍需进一步转变。等等。对此，市政府将在今后工作中采取切实措施，认真加以解决。

回顾过去三年的工作，我们深深体会到，要实现我市国民经济和社会各项事业的加快发展，必须始终坚持以邓小平理论和“三个代表”重要思想为指导，认真落实科学发展观，求真务实，开拓进取，结合实际创造性地开展工作；必须以加快经济建设为中心，抢抓发展机遇,尤其要抓住世界范围内和省内产业转移机遇，推进全市经济社会加快发展；必须以工业化为龙头，带动城镇化和农业产业化发展，不断优化全市经济结构；必须加大招商引资力度,加快工业园区建设步伐,狠抓项目建设，增强工业发展后劲；必须加快改善交通、城建、能源等基础设施，为经济加快发展创造良好的环境条件；必须调动各方面积极性，形成加快发展合力，促进市、县、镇三级经济全面发展，壮大全市经济总量；必须统筹城乡协调发展，加快发展农村经济，不断改善农村的生产、生活环境，建设社会主义新农村；必须坚持统筹经济社会发展，结合云浮实际，创造条件，大力推进科技、教育、文化、卫生、体育等各项社会事业，促进全市经济社会协调发展；必须自觉加强政府自身建设，为全面履行政府职责提供强有力保障；必须加强和谐社会建设，解决好民生问题，及时化解各种社会矛盾和群众纠纷，坚决打击各种犯罪活动，为实现经济社会快速协调发展提供良好的社会环境。

今后五年的奋斗目标和总体要求

各位代表，当前我国政治稳定，经济快速发展，已经成为经济发达国家、地区产业转移和资金的重要流入地；我省与港澳台及泛珠三角地区各省的经贸合作关系日趋紧密，珠三角产业向山区和东西两翼转移的势头更加明显；我市经过建市十二年的发展，形成了一批特色产业和一定的经济基础，明确了加快经济社会发展的路子，初步积累了加快山区经济强市与和谐云浮建设的经验和体会。我市已初步具备了加快发展的良好机遇和基础条件。因此，未来五年，是承前启后、继往开来的五年，是我市建设山区经济强市、构建和谐云浮关键的五年，是抢抓机遇、迎接挑战、加快发展的五年。市政府将在市委的领导下，团结和带领全市人民，艰苦奋斗，狠抓落实，努力开创我市经济建设和社会发展新局面。

根据市第四次党代会精神，今后五年政府工作指导思想是：高举邓小平理论和“三个代表”重要思想伟大旗帜，牢固树立和认真落实科学发展观，继续实施“东渐西联、优内拓外、错位竞争、统筹并进”发展战略；坚持走工业化、城镇化、农业产业化发展路子，加快新型工业化发展步伐，调整和优化经济结构，促进一、二、三产业协调发展；加快县域、镇域经济发展，增加二、三产业在县镇经济中的比重，促进市、县、镇三级经济协调发展；加快发展科技、教育、文化、卫生、体育等各项社会事业，促进经济社会协调发展；坚持依法治市、依法行政，促进物质文明、精神文明和政治文明协调发展；坚持以人为本，注重民生和社会公平，促进人的全面发展和人与自然的协调发展，为实现建设山区经济强市、构建和谐云浮的宏伟目标打下坚实基础。

今后五年政府工作的奋斗目标是：全市生产总值和财政一般预算收入年均增长分别达到13%和15%左右，力争到2011年比2006年实现翻一番，到2013年实现省委要求比2003年翻两番的目标，主要经济指标增幅高于全省平均水平，全市综合经济实力走在全省山区市前列，初步建成经济实力更加壮大、城市特色更加鲜明、生态环境更加优化、和谐程度更加提升、幸福指数更加提高的新云浮。

为此，今后五年，我们一要始终坚持以科学发展观为指导，把加快发展，尤其是加快工业发展作为经济工作重中之重，不断增强全市整体经济实力。坚持走新型工业化道路，实施科技带动、名牌带动和外向带动战略，加大招商引资和承接产业转移工作力度，加强现有企业改造和产业提升，发展一批产业集群，加快工业园区建设，抓紧抓好大型骨干项目的申报、立项和建设工作，不断提升工业化水平；继续大力推进县域、镇域经济发展，加快发展现代农业和服务业步伐，提高民营经济水平，扩大全市经济规模。二要始终坚持按照“生产发展、生活宽裕、乡风文明、村容整洁、管理民主”的要求，稳步推进社会主义新农村建设。加快发展农村经济，提升农业产业发展水平；加强规划与管理，建设农村新村貌；培育现代新农民，树立农村新风尚；探索改革新机制，推进“活力民主、阳光村务”建设。三要始终坚持把优化发展环境摆在重要位置，加快改善经济社会发展环境条件。加快交通、城建、能源、通信、水利等基础设施建设，增强中心市区、县城和中心镇的区域辐射带动能力，进一步改善投资硬环境。强化政府创造发展环境的主体地位，努力建设法治政府、服务政府、责任政府、效能政府和廉洁政府，营造良好的政务环境、商务环境、创业环境、治安环境和城市环境。四要始终坚持统筹发展各项社会事业，促进人的全面发展和人与自然的协调发展。注重社会建设，提高科技、教育、文化、卫生、体育、人口与计划生育等社会事业发展水平；加强生态建设，发展生态经济，加强环境保护，努力构建宜居宜业环境。五要始终坚持以人为本，为民谋利的施政理念，全面推进和谐云浮建设。关注民生民情，认真做好与人民群众利益密切相关的劳动就业、社会保障、职工工资、扶贫救灾等各项工作，让群众共享改革发展成果；推进依法治市，坚持和完善各项民主政治制度，保障社会公平正义；加强社会管理，维护社会大局和谐稳定。

各位代表，展望未来，我们充满信心，豪情满怀。我们坚信，只要我们求真务实，积极进取，扎实工作，就一定能够让我市的经济发展得更快，让群众生活得更好，让社会环境更加和谐，就一定能够圆满实现今后五年经济社会发展各项目标任务。

2007年的工作意见

各位代表， 2007年是新一届政府工作的开局之年，各项工作要努力实现新发展，取得好成绩，为实现今后五年的工作目标打下更加坚实的基础。2007年全市经济和社会发展的预期目标是：生产总值比上年增长13.5%，其中第一产业增长5%、第二产业增长23%、第三产业增长15%，单位生产总值能耗下降3%，社会消费品零售总额增长17%，全社会固定资产投资增长20%，地方财政一般预算收入增长18%，外贸出口总额增长25%，实际利用外资增长30%，农村居民人均纯收入增长6%，城镇居民人均可支配收入增长10%，人口自然增长率控制在7‰以下，城镇登记失业率控制在3%以内,居民消费价格总指数控制在2%以内。

实现今年经济社会发展预期目标，必须做好以下十方面工作：

（一）加快新型工业化发展步伐，进一步提高工业化水平，促进全市经济结构优化。

结合资源条件和产业特点，加快推进新型工业化进程，进一步加大招商引资力度，积极承接产业转移，发展民营经济，推进国有企业做大做强，加快项目和园区建设步伐，大力发展产业集群，努力提高全市工业经济总量和质量。加快发展工业产业集群，进一步做大做强我市现有工业产业。认真落实各项政策措施，做大做强石材、不锈钢制品等省级产业集群，同时积极引导硫化工、水泥、纺织服装、机械电子及农产品深加工等行业的企业加强生产、销售、技术合作，实现资源集约和优势互补，培育壮大新的产业集群；积极利用产业集群的竞争优势和“洼地”效应，引进关联企业，壮大产业集群规模和产业配套能力；积极引导工业龙头企业实施名牌带动战略，多创名牌产品和著名、驰名商标，提高自主创新能力；继续推进建材、硫化工、农林产品深加工等产业延伸产业链，带动上下游配套产业，推进这些产业集群发展。大力度推进珠三角产业转移的承接工作。进一步加强我市与佛山等珠三角地区各级政府和工商企业界的合作，巩固和发展近两年来产业转移合作成果；主动走出去，不断扩大承接产业转移的领域；加强领导，各级政府主要领导要亲自协调解决承接产业转移工作中的困难和问题，积极主动做好相关配套服务工作。积极推进项目和园区建设。完善重点项目建设台帐管理制度，加强已签约项目的跟踪落实。争取云浮电厂三期工程尽快获国家发改委核准并动工建设、华润西江发电厂通过国家电力项目评优并于2008动工建设、西江内河核电郁南罗旁厂址尽快进入可行性研究。加快推进云硫集团40万吨硫酸和天山、中材亨达水泥二期等扩建项目以及亚铝集团投资项目建设，开展粤泷电厂二期等大项目论证工作。加快推进都杨、新成产业转移工业园，思劳高新区以及各县（市、区）工业园区的规划建设步伐，完善水、电、路、环保、信息化等配套设施建设。加大园区招商推介力度。都杨、新成两个园区年内要有一批项目进园建设。积极推进循环经济发展。以电力、水泥、硫化工、石材、冶炼、不锈钢制品等高耗能行业为重点，加快推广应用资源节约和综合利用技术，推行清洁生产。加快推进创建循环经济示范县和环保工业园建设，大力引进节约土地、综合利用资源的二、三产业项目。积极做好协调服务工作。各级政府部门要进一步转变机关工作作风，落实工作责任制，完善抓落实工作机制，不断提高机关工作效率和质量；经常深入企业，了解和帮助企业解决生产中煤、电、油、运、用地、用工和资金等困难，保证企业正常生产；积极加强与上级主管部门的沟通联系，争取并落实相关产业政策，充分发挥政府部门服务工业发展效能；积极协调企业产品质检工作，完善出口通道建设，降低进出口贸易成本；加快推进与高校、科研院所的产学研合作，为企业提供科技动力和智力保障。

（二）加快农业和农村经济发展，千方百计增加农民收入，扎实推进社会主义新农村建设。

建设社会主义新农村，关键是加快农村经济发展。坚持用现代发展理念引领农业，加大工业反哺农业、城市支持农村的力度，深化农村综合配套改革，在确保粮食安全的基础上，加快全市农村经济发展。大力推进农业产业化经营。继续引导农业龙头企业与农业商品生产基地相结合，大力发展农林产品深加工业，提高农产品附加值；引导农民参与和发展专业合作经济组织，继续发展订单农业，提高农业商品生产基地建设水平，打造优势农产品产业集群。继续推进石灰岩地区农业产业化经营，千方百计增加苦旱地区农民收入。大力推进现代农业建设。加强基本农田保护和农业基础设施建设，实施农业综合开发。年内改造中、低产田4.9万亩。继续抓好云城、新兴农业现代化功能区和全市23个优势农产品示范基地建设。加大农产品生产环节的监管,大力推进农业标准化生产，发展无公害、绿色、有机、健康食品，积极创建农产品商标和地理标志，打造农产品品牌。加大重大动植物疫病防控。大力发展山区特色农林产业，提高农林产品经济效益。继续发展富有山区特色的畜牧、水果和蔬菜、优质稻、蚕桑、南药、松脂、肉桂、花卉和城市绿化苗木等农林产业。拓宽农林产品流通渠道，充分发挥粤西泷州农产品交易中心集散作用，加快思劳农产品交易市场建设。大力发展劳务经济，增加农民非农收入。建立促进农村劳动力有序转移服务体系、培训体系和市场体系，引导农村富余劳动力向非农产业和城镇转移就业；充分发挥技校、职中以及各类培训中心培训农民工的作用，推广订单式培训，重点开展电子、纺织服装、建材、不锈钢制品、农林产品深加工等行业和各类服务业的用工培训，以及农民种养技能培训；继续实施“智力扶贫”工程，增强贫困地区群众自我发展和到非农产业就业能力。

（三）加快服务业发展，壮大经济发展新增长点。

逐步提高服务业在全市经济中的比重，是优化我市经济结构、提高经济质量的重要内容，也是拉动经济增长、扩大就业的潜力所在。加快发展旅游业。从政策、资金、技术和人才等方面加大对旅游业的扶持力度。完善旅游发展规划，充分利用自然生态、人文历史、优质温泉等资源条件开发旅游项目，打造旅游品牌，发展特色旅游。利用民资和引进外资加快一批档次较高的旅业和餐饮业设施建设，提高城市服务能力和城市品位。积极推进创建中国优秀旅游城市活动，力争年内创优工作通过国家验收。加快发展商贸业。加大西江航运的开发力度，继续推进档次较高的商贸区和专业市场建设，培育物流、连锁经营、信息、中介和社区服务等新兴行业，加快改善全市交通运输和商贸、服务条件。继续推进“万村千乡”市场工程，加快农村市场建设。积极发展会展经济，依托本地特色产业和名优产品，通过举办各类产业展销、技术博览、宣传推介活动，逐步打造面向全省、全国乃至世界的会展平台，加快商贸扩张，拉动产业发展。加快发展房地产业。继续落实中心镇以上城镇规划区不再批地建单门独户私房措施，推进市区、县城、中心镇住宅小区建设，繁荣房地产市场。

（四） 加快县域、镇域经济发展步伐，推进全市区域协调发展。

大力协调区域发展，进一步做强县域经济。坚持以工业化为龙头，走工业化、城镇化、农业产业化发展路子。各县（市、区）都要积极发挥业缘、地缘和资源优势，做大做强石材、硫化工、不锈钢制品、水泥、纺织服装、电子电器、农产品深加工等产业，继续推进农业产业化经营，积极拓展服务业，加快经济发展。云城区要发挥中心城区优势，突出发展二、三产业，壮大城市型经济，增强对全市的经济辐射力；新兴县要利用较好的区域条件和产业基础，做好承接产业转移、发展产业集群、养殖业和旅游业等工作；云安县要继续打造硫化工、水泥、石材循环经济产业链，建设硫化工和建材产业基地；罗定市、郁南县要努力改善交通等基础设施条件，加快工业园区建设，优化投资环境，在做大原有纺织服装、机械、电池、电子等产业的基础上，利用资源优势，扩大招商引资，努力上一批新项目，加快经济发展速度。继续推进镇域经济发展。各镇（街）要从实际出发，坚持宜工则工、宜农则农、宜旅游则旅游。大力发展自然资源、农林产品深加工业和劳动密集型加工业。认真研究和落实省扶持山区县、中心镇、专业镇发展的政策措施，加快城镇基础设施和公共设施建设。进一步加强招商引资，承接产业转移工作。加快推进农村工业化、农业产业化、特色农业发展。继续大力发展民营经济。认真落实国家和省、市加快民营经济发展的优惠措施，放宽准入条件和领域，创造民营经济放开手脚、大力发展的宽松环境。加强对民营企业主的培训，组织企业主走出去开拓市场、开阔眼界，增强干事创业能力和经营管理水平。积极做好用地、用水、用电、用工、人才引进、科技引进、创立品牌等服务工作。坚持增加数量和提高质量并举，各县（市、区）都要大力培育一批民营龙头企业。

（五）继续强化招商引资和外经贸工作，增强全市经济发展后劲。

进一步加大招商引资工作力度。抢抓省内、国内以及国际产业转移机遇，加快引进项目、资金、技术、人才和设备，进一步推进我市二、三产业加快发展。各级政府要积极搭台，主要领导要亲自组织实施招商活动，带领企业主动走出去，吸引市外产业转移项目到我市落户；充分利用我市产业和资源优势，加强资源、产业和园区的宣传推介，充分发挥政协委员、海外社团和行业协会（商会）以及驻珠三角招商机构等的桥梁作用，促进招商引资企业化、专业化、社会化。既要争取引进大企业、知名企业，又要重视引进中小项目，还要鼓励现有外资企业增资扩产。扩大对外经济发展空间。进一步落实国家、省扩大出口的各项政策，鼓励和支持民营企业申报自营出口经营权和免检产品。实施科技兴贸和品牌战略，优化出口商品结构，扩大高新技术产品、机电产品出口，扶持农产品深加工出口，提升纺织服装、不锈钢制品、石材等产品出口档次。着力加快口岸建设，加快推进石材和不锈钢制品两个保税仓建设。巩固港澳台、欧美日等传统市场，开拓拉美、中东、东盟等新市场，拓展发展空间。组织协调好第四届中国（云浮）国际石材科技展览会，积极参加省组织的有关经贸活动。积极扩大生产设备、原材料、能源等物资和其它生产生活资料的进口，加快做大我市外经贸产业。

（六）继续强化财税金融工作，保障经济社会稳健运行。

强化以人为本的公共财政理念，加强财税金融工作，增强对经济社会发展的调节、保障和促进作用。努力增加财税总量，提高财税质量。加快二、三产业发展和重点项目建设，建设支撑有力、后续旺盛、结构合理的财源体系。严格依法治税，努力打造诚信纳税环境，着力提高税收征管的质量、效率和水平。继续推进政府资源的管理和开发，进一步规范非税收入管理，确保财政收入持续稳定较快增长。科学安排财政支出。完善公共财政制度，优化支出结构，加大财政对社会主义新农村、科技、教育、文化、公共卫生、就业、社保、社会治安、环境保护和生态建设等方面的投入。规范部门预算、政府采购、国库集中支付、财政支出绩效评价制度。加强国有资产管理。控制政府举债规模，防范财政风险。深化预算执行审计、效益审计和经济责任审计，提高财政资金和国有资产的使用效益。积极改善融资环境。进一步加强银企合作，吸引更多金融机构落户云浮。积极盘活社会闲散资金，支持企业到资本市场融资。稳步推进农村信用社改革，扎实抓好城信社、农金会的资产追收工作。加强金融监管，严厉打击洗钱活动。支持商业保险实行公平竞争，开发新险种，发展新业务。

（七）继续强化基础设施建设，加快改善发展环境。

加快交通网络建设。积极配合业主单位加快广梧高速公路二期工程建设、云浮至广西岑溪高速公路（粤境段）和云浮至新兴段高速公路立项工作，加快云安六都至高要大湾沿江公路建设进度，开展河杨公路扩建和国道324线罗梅段改造申报立项工作。加快农村公路、罗定至岑溪段铁路、云浮港六都四围塘和都骑通用码头的建设进度。年内全市交通建设计划总投入17.3亿元。加快城镇化建设。年内完成市区兴云中路、兴云西路、城中路、建设路等主要道路改造，加快西花岗市政综合开发。继续推进市委党校新校区、市迎宾馆三期、市图书馆、博物馆和市老干部活动中心等项目建设。按市管镇、县管村的要求，加强城乡规划管理，完成城区总体规划及“十一五”近期建设规划修编，完善县城和中心镇规划，加快供水、供电、环保和卫生等市政公共项目建设。加强社区管理，优化居住环境，引导农村人口向城镇集聚，加快城镇化进程。年内全市城镇建设计划总投入12.7亿元。加快能源、水利和公共卫生设施建设。继续推进能源项目申报建设工作，改造完善全市城乡电网和输变电设施。动工兴建市区南山河二期、郁南县城综合防洪等3宗城乡水利防灾减灾工程，争取在年内全面完成5宗城乡水利防灾减灾工程和23宗小型水库除险加固等工程，解决23条水库移民村的遗留问题；推进城乡群众饮水安全工程建设，年内争取解决全市26万群众的饮水安全问题。年内全市水利建设计划总投入2.8亿元。加快新兴、郁南生活污水处理厂等10个环保项目建设，年内建成4个。年内全市环保建设计划总投入2.2亿元。完善城区供水应急工程。加快信息通信基础网络建设，实现网络到镇、信息进村。加快政务信息化、企业信息化和社会领域信息化步伐。大力发展通信事业。

（八）坚持发展各项社会事业，促进社会全面进步。

加快发展科技事业。切实提高与高等院校和科研院所合作水平，积极引进高新技术改造传统产业，加快建材、机械设备、农产品深加工、禽畜和水产养殖等科研成果的转化。年内要争取组织实施省以上重点科技项目15项，申报专利150项、高新技术企业2个、民营科技企业4个、专业镇3个。加快发展教育事业。积极开展规范化学校建设，巩固和发展“一机两语”特色教育，推进城乡义务教育均衡发展。大力发展普通高中教育和职业教育，提高高中阶段教育普及率。深化义务教育经费保障体制改革，在全市农村全面实施免费义务教育。鼓励社会力量兴办职业教育。推进市技工学校、市中等职业技术学校建设。努力建设高素质的教师队伍。切实加强新时期青少年学生思想道德建设，全面推进素质教育。年内全市普通和职业教育计划总投入12亿元。加快发展文化事业。坚持一手抓繁荣，一手抓管理，努力丰富群众文化生活。培育文化市场，发展文化产业，努力打造南江文化、禅宗文化、石文化等特色文化品牌。抓好非物质文化遗产保护工作。加快推进有线数字电视整体转换步伐，年内实现全市有线电视光纤联网到镇，争取尽快实现“村村通”。继续开展学习型机关、学习型企业、学习型社区创建活动，努力提高市民素质。加快发展卫生事业。继续完善疾病预防控制、卫生监督执法和医疗救治三大体系建设，年内完成改造10间薄弱卫生院，完善842个村卫生站，落实乡村医生补助政策；稳步推进医疗卫生体制改革，规范医疗服务项目、收费以及药品和医疗器械集中招标采购工作；加强对禽流感、狂犬病和艾滋病等重大疫病的监测。加强农村卫生工作，巩固和完善新型农村合作医疗制度，发展中医药事业，加快创建农村中医工作先进市。继续推进卫生城市创建工作。加快发展体育及其它各项事业。加强群众性体育设施建设，广泛开展全民健身运动，提高竞技体育水平，办好第三届市运会。完善人口和计划生育目标管理责任和利益导向机制，综合治理出生人口性别失衡，开展创建计生优质服务先进县和“两无”（无政策外多孩出生镇街和无政策外出生村）活动，确保完成省下达的人口和计划生育目标任务。广泛深入开展全民国防教育，大力抓好民兵预备役建设。继续推进创建双拥模范城，争取年内通过上级验收。完成市直部门志书修编和《云浮市志》初稿。做好人事人才、民族、宗教、外事、侨务、台务、统计、民政、妇儿、气象、人防、档案、红十字会等工作。

（九）坚持以人为本，努力解决民生问题，不断推进和谐云浮建设。

在加快经济发展的同时，高度重视人民群众的利益协调和保障工作，努力实现人与人和人与自然、资源、环境和谐共存，协调发展。切实解决群众关心的热点、难点问题。加快农村劳动力转移培训基地建设，继续推进“百万农村青年技能培训工程”，年内实施培训3万人；抓好退伍军人免费技能培训。统筹做好国有企业下岗失业人员、大中专学校和职业技术学校毕业生、复退军人以及城乡新增劳动力转移就业工作；强化社会保险扩面征缴，确保各项待遇按时足额发放；加强劳动保障监察工作，切实维护劳动者合法权益；加强妇女儿童、老年人、残疾人权益保障工作；完善城乡最低生活保障制度，完善五保供养工作，建立补助金自然增长机制；扩大廉租住房保障范围,确保城镇特困居民居有定所，力争年内全部解决农村住房难、行路难问题；着力解决被征地农民就业和生活出路问题；继续加强农民减负工作监管，确保农民负担不反弹；继续抓好水库移民后续援助工作。切实抓好环境保护和生态建设。继续加强西江、南江、新兴江、南山河综合治理，切实保护饮用水源。建立健全环境监督执法、环境监测体系，严格执行建设项目环保准入制度，严格控制主要污染物排放总量，严厉查处违法排污行为。加强森林资源保护，推进林业生态县和林业生态市建设。加大村庄整治力度，年内建设生态文明村600条，发展农村沼气池5000个。加强地质灾害调查、监测、防治和防震减灾工作，提高气象预警预报能力。切实抓好社会稳定与平安云浮建设。大力加强公安司法机关基层基础建设，落实人员、装备、经费动态保障机制，加强社会治安综合治理，加快推进镇（街）综治中心建设；严厉打击 “两抢一盗”等多发性侵财犯罪、有组织犯罪、毒品犯罪以及严重暴力犯罪；开展平安市、县创建活动，推进城市社区和农村治安防范工作；深入开展普法工作，提高公民法律素质；加强人民调解工作，努力化解人民内部矛盾；完善领导接访、领导包案、信访工作目标管理考核和责任追究等制度，切实解决群众信访反映的热点难点问题，预防和处置群体性突发事件。切实抓好安全生产。认真落实安全生产责任制。大力推进安全生产监管制度建设。重点抓好危险化学品、烟花爆竹、非煤矿山、道路与水上交通、消防和建筑等安全专项整治工作，以强有力措施坚决遏制和防范重特大事故的发生。

（十）坚持推进民主法制和政府自身建设，提高政府管理效能。

为履行好新一届政府职能，必须进一步强化自身建设，努力建设法治政府、服务政府、责任政府、效能政府和廉洁政府。强化民主法制建设，促进民主科学决策。 坚持向人大及其常委会报告工作，自觉接受人大及其常委会的监督，认真落实人大及其常委会的决议、决定。主动向人民政协通报情况，接受民主监督。认真办好人大代表议案、建议和政协提案。广泛听取各民主党派、工商联、无党派人士的意见。充分发挥工会、共青团、妇联、侨联、残联、红十字会等群团组织的桥梁纽带作用。全市经济社会发展战略、重要发展规划、重大建设项目以及关系民生的重要决策，要坚持走群众路线，广泛听取各方面的意见，进行充分论证，提高科学决策水平。强化依法行政，加强经济社会管理。加强对法制工作的领导。进一步完善政府各项工作规章制度。严格依照法定权限和程序行使权力，履行职责，接受监督。加快社会信用体系建设，严惩制假售假、商业欺诈、走私贩私、偷逃骗税、金融诈骗和侵犯知识产权等行为，严厉打击商业贿赂及传销和变相传销活动。加强生产、流通等环节的安全监管，让人民群众吃上放心食品、药品。建立健全各种突发事件应急机制，提高保障公共安全和处理突发事件能力。加快转变政府职能，积极支持行业协会（商会）开展维护企业权益、推动科技进步、开拓产品市场等工作。强化政务监督，狠抓工作落实。继续推进政务公开，通过行风评议、政风行风热线、市长热线、城市论坛等形式加强舆论与社会监督。加强行政监察和行政复议，完善行政监督制度，努力提高政府部门工作效率和质量。严格实行执法责任制和执法过错追究制，深入开展廉政建设，切实做到有权必有责、用权受监督、侵权须赔偿、违法要追究。进一步规范政府采购、政府投资工程建设、经营性土地使用权和采矿权有偿出让及国有、集体资产进场公开交易制度。完善政府分解年度工作要点、部门分工抓落实新机制，使各项工作有人组织、有人落实、有人检查验收，确保取得实际效果。切实加强公务员队伍教育和管理，不断强化大局意识、发展意识、机遇意识、责任意识和实干意识，努力建设政治坚定、业务精通、清正廉洁、作风优良的公务员队伍。

各位代表，当前我市改革、发展和稳定工作任务繁重、责任重大、使命光荣。让我们高举邓小平理论和“三个代表”重要思想伟大旗帜，更加紧密地团结在以胡锦涛同志为总书记的党中央周围，全面落实科学发展观，求真务实，开拓创新，全力推动经济社会加快发展，努力实现建设山区经济强市、构建和谐云浮的宏伟目标！
